# Supplementary material for: Heart rate variability analysis for the assessment of immersive emotional arousal using virtual reality: Comparing real and virtual scenarios
Source: PLoS One. 2021 Jul 1;16(7):e0254098. doi: 10.1371/journal.pone.0254098 (PMC8248697; doi:10.1371/journal.pone.0254098)
Supplement: Attachment — Submitted filename: Response to Reviewers.docx [file pone.0254098.s002.docx]

**Statement of Changes and Answers to Comments**

We would like to thank the academic editor and reviewer for their careful consideration of our paper and for their valuable suggestions as to how to improve it. We believe that this revised version of manuscript ref. number PONE-D-20-32832R1 addresses the criticisms/comments raised by the reviewer. The revised text is highlighted in red to easy the revision process.

**Answer to reviewer**

**Reviewer #2**

**Comments:**

*Q2.1)	The authors have addressed most of my suggestions in this revision. There are two issues which remain. First, although there have been several changes to reference "arousal" rather than "emotion", i think a change in the title of the paper to this effect is also necessary.*

R2.1)	Thank for your comment. Following the reviewer suggestion, the title has been changed to “Heart rate variability analysis for the assessment of immersive emotional arousal using virtual reality: Comparing real and virtual scenarios”

*Q2.2)	Second, my previous comment about validation (Q2.4 in the response to review) was not about validation of the model per se, which I understand was likely confusing due to my comment on AUC. Rather I'm hoping the authors can explain how their paper addresses the comments they raise about the "validity" of VR, such as in the abstract in line 22 and also in line 121. What does it mean that VR needs to be validated and how does the current analysis address this.*

*R2.2)*	Thank you for your comment. Many researchers are currently using VR to replicate tasks to assess human behaviour, therefore a thorough investigation on the validity of those environments, and in particular the similitudes and differences between VR and physical environments, is critical.

The concept of validity is presented in the introduction (line 121) and refers to the capacity of VR to evoke a response from the user equal to the one that might be evoked by a real physical environment.

According to such definition, the present work investigates VR validity in the emotional arousal dimension and shows that, while the direct virtualisation of a real environment might be self-reported as evoking psychological arousal, it does not necessarily evoke the same cardiovascular changes as a real arousing elicitation.

We believe this is an important finding, which might shed light to future VR and emotional research.
